# A Scientometric Evaluation of the Chagas Disease Implementation Research Programme of the PAHO and TDR

**DOI:** 10.1371/journal.pntd.0002445

**Published:** 2013-11-07

**Authors:** Ana Laura Carbajal-de-la-Fuente, Zaida E. Yadón

**Affiliations:** 1 Leishmaniasis Transmitters Laboratory, Oswaldo Cruz Institute, FIOCRUZ, Rio de Janeiro, Brazil; 2 Laboratory of Eco-Epidemiology, Department of Ecology, Genetics and Evolution, (IEGEBA-CONICET) University of Buenos Aires, Buenos Aires, Argentina; 3 Communicable Diseases Unit, Health Surveillance, Disease Prevention and Control, Pan American Health Organization, Washington, D.C., United States of America; René Rachou Research Center, Fiocruz, Belo Horizonte, Brazil, Brazil

## Abstract

The Special Programme for Research and Training in Tropical Diseases (TDR) is an independent global programme of scientific collaboration cosponsored by the United Nations Children's Fund, the United Nations Development Program, the World Bank, and the World Health Organization. TDR's strategy is based on stewardship for research on infectious diseases of poverty, empowerment of endemic countries, research on neglected priority needs, and the promotion of scientific collaboration influencing global efforts to combat major tropical diseases. In 2001, in view of the achievements obtained in the reduction of transmission of Chagas disease through the Southern Cone Initiative and the improvement in Chagas disease control activities in some countries of the Andean and the Central American Initiatives, TDR transferred the Chagas Disease Implementation Research Programme (CIRP) to the Communicable Diseases Unit of the Pan American Health Organization (CD/PAHO).

This paper presents a scientometric evaluation of the 73 projects from 18 Latin American and European countries that were granted by CIRP/PAHO/TDR between 1997 and 2007. We analyzed all final reports of the funded projects and scientific publications, technical reports, and human resource training activities derived from them. Results about the number of projects funded, countries and institutions involved, gender analysis, number of published papers in indexed scientific journals, main topics funded, patents inscribed, and triatomine species studied are presented and discussed.

The results indicate that CIRP/PAHO/TDR initiative has contributed significantly, over the 1997–2007 period, to Chagas disease knowledge as well as to the individual and institutional-building capacity.

## Introduction

Chagas disease or American trypanosomiasis is caused by the protozoan *Trypanosoma cruzi* (Chagas, 1909) and transmitted to humans by a group of hemipteran insects belonging to the family Reduviidae, subfamily Triatominae [1]. The main mode of transmission in endemic areas is vectorial, by domestic, peridomestic, or sylvatic triatomines. The disease can also be transmitted by the congenital and oral routes, blood transfusion, organ transplantation, and laboratory accidents [2]. Currently, Chagas disease is considered a serious health problem in Latin America, with 9–10 million people infected [3]. It extends from the southern United States to southern Argentina and Chile [4], although it has been shown [5] that migration from endemic American countries to nonendemic countries makes Chagas disease a global health problem.

In 1984, it was estimated that over 100 million people in Latin America were at risk and that 24 million were already infected with *T. cruzi* [6]. Great progress has been made in its prevention and control in Latin America since the 90 s. Transmission has been interrupted in territories of the Southern Cone, Central American, Andean, and Amazonian Intergovernmental Initiatives led by the Pan American Health Organization (PAHO) [3]. These initiatives have led to substantial reductions in vectorial and transfusional transmission, decreasing the number of infected people from 16–18 million in the 90 s to 9–10 million currently [2], [4]. These advances and the interruption of *T. cruzi* transmission in many endemic countries have been possible because of the strong commitment of the endemic Member States, the strength of their national research and control organizations, and the support from many international partners [4], including Japan International Cooperation Agency (JICA), Canadian International Development Agency (CIDA), European Community-Seventh Framework Programme (EC-FP7), European Community-Latin American Network for Research on the Biology and Control of Triatominae (ECLAT), World Vision (WV), CARE International (CI), *Médecins Sans Frontières* (MSF), and the Special Programme for Research and Training in Tropical Diseases (TDR), among others [7]–[9].

Chagas disease research and control programmes are included in both the portfolios of the Communicable Diseases Unit/PAHO (CD/PAHO) and TDR. TDR is an independent global programme fostering scientific collaboration [10] that was established in 1975 to address the need for new and improved tools for disease control, and for strengthening the research capacity of endemic countries [11].

In 2001, in view of the achievements obtained in the reduction of the transmission of *T. cruzi* in the Southern Cone and the improvement of vector control activities in some countries of the Andean and the Central American Initiatives, TDR decided to transfer CIRP/PAHO/TDR to CD/PAHO [12], [13]. As a result, CD/PAHO also received the responsibility of managing the ongoing projects approved by TDR from 1997 to 2001. The first PAHO call for proposals was in 2002. One and a half million dollars to support research, training activities, and managerial issues were allotted for the 2002–2008 period.

To ensure that all research supported by CIRP/PAHO/TDR was conducted to the highest scientific level, a peer-review process was established, led by a multidisciplinary, multicountry, and gender-balanced Steering Committee (SC).

The evaluation of results achieved by a research programme is essential for formulating, reviewing, and improving institutional research policies aimed at ensuring the appropriate use of financial, human, and material resources and to promote strengthening and growth of an institution [14], [15]. In order to evaluate the research impact of CIRP/PAHO/TDR, we performed a scientometric analysis of its scientific and capacity-building achievements for the 1997–2007 period.

## Material and Methods

The relevant projects for this analysis were identified through a database developed by PAHO that included information about investigators, home institutions, and key data from new, progress, renewal, and final scientific reports to TDR. In addition, relevant data used for the scientometric analysis were retrieved from the following specific sources:

### 1. Scientific Reports

Applicable data were extracted from the projects' scientific reports approved by the TDR Task Force from 1997 to 2001 and by the SC of CIRP/PAHO/TDR from 2002 to 2007. The scientific reports contained the main scientific contributions, publications, training, and patents derived from the research projects for the reporting period.

### 2. Publications Databases

Two databases were searched for the scientific production on Chagas disease research for the 1997–2010 period: PubMed (http://www.pubmed.com) and LILACS (http://lilacs.bvsalud.org/en/).

We searched for articles published in English, Portuguese, and Spanish that presented results of studies granted by CIRP/PAHO/TDR. The search terms used were the principal investigator (PI) name and the keywords related to the research subject. The number of citations of each article was obtained from a search in the Web of Knowledge databases. The five-year impact factor and the ISSN of each scientific journal were extracted from the Journal Citation Reports of the Web of Knowledge.

We also searched PubMed using the term "triatom*" in order to determine the ratio of publications derived from projects granted by CIRP/PAHO/TDR to the overall Chagas disease scientific production.

### 3. In-Depth Survey

Each PI filled a structured questionnaire to provide additional information on the publications (articles published in scientific journals, meetings proceedings, books, or book chapters; abstracts or expanded meetings abstracts; and texts in newspapers or magazines) related to the projects granted by the CIRP/PAHO/TDR. The questionnaire included additional questions about technical assistance (technical consultations and reports for governmental health authorities), extension activities (community workshops or other activities involving schools, communities, or health agencies, dissemination materials such as promotional materials, booklets, CDs/DVDs, radio and TV programmes, websites, etc.), and individual and institutional capacity building (graduation, specialization, Master and Ph.D. degrees, postdoctoral fellowships, and capacity-building or training activities).

## Results

### 1. Scientific Reports Analysis

A total of 73 projects were included in the present study: 16 projects transferred by TDR to CD/PAHO and funded between 1997 and 1999; 14 new projects funded in 2000–2001; and 43 projects funded between 2002 and 2007 (13 in 2002; ten in 2003; 12 in 2005; four in 2006; and four in 2007).

**Figure 1:**
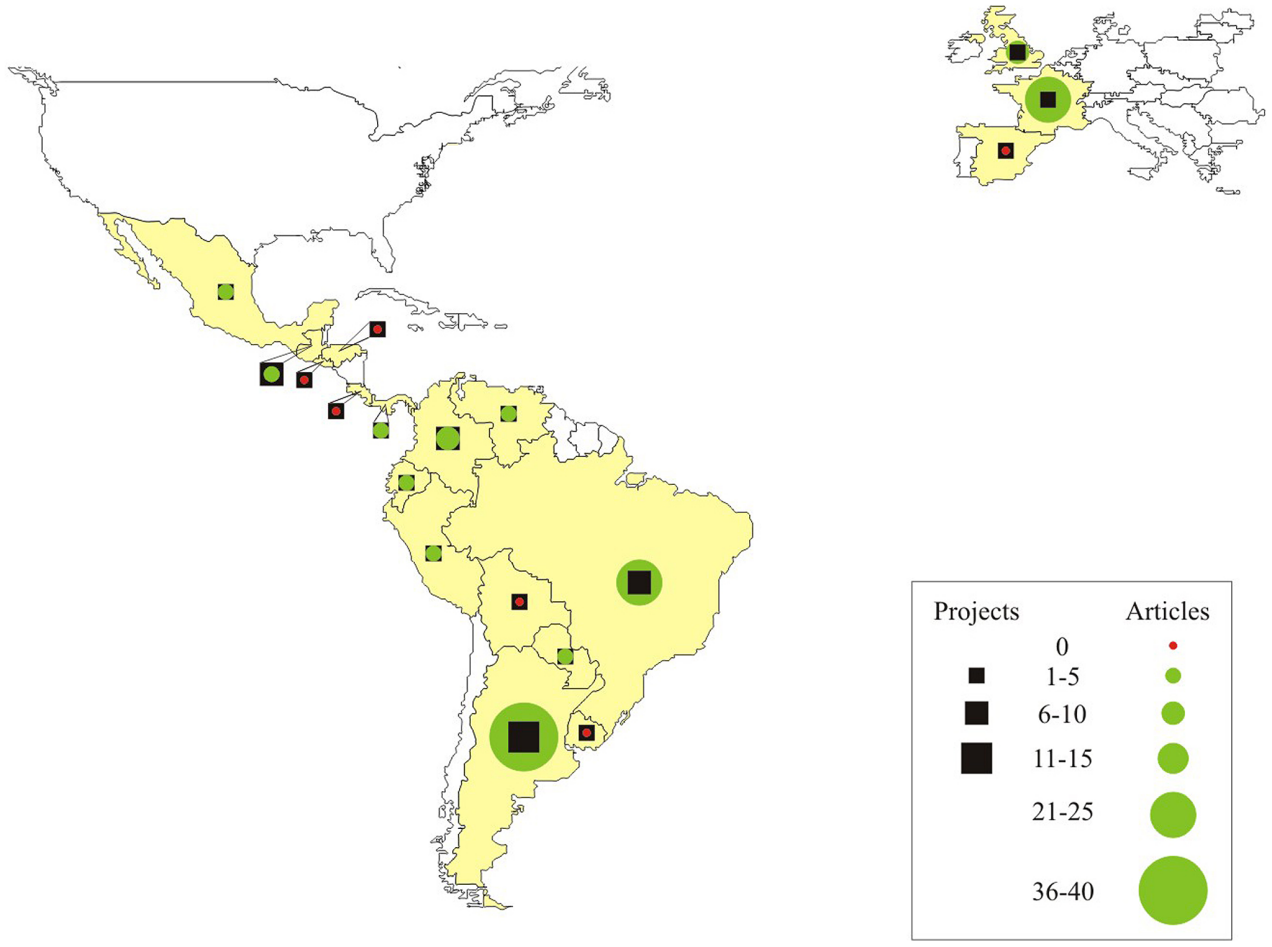
Projects funded and proportion of published articles in indexed scientific journals by countries, CIRP/PAHO/TDR (1997–2007).

The 73 projects were coordinated by 58 PIs from 18 Latin American and European countries (Figure 1). The highest number of projects carried out by one single PI was five. PIs of 59% (n=43) of the projects were male, and 41% (n=30) were female. Six countries (Argentina, Mexico, Colombia, Ecuador, Brazil, and France) had both male and female PIs, four (Guatemala, Paraguay, Venezuela, and Uruguay) had only female PIs, and eight (Bolivia, Costa Rica, El Salvador, United Kingdom, Honduras, Panama, Peru, and Spain) had only male PIs.

The majority of the institutions involved (78%) were located in developing countries. However, notable asymmetry in the number of funded projects per country was noted, with 46% of the projects concentrated in 22% of the participating countries. Argentina had the highest number of PIs granted (n=13), followed by Colombia (n=8), and Brazil (n=7). Argentina also had the highest number of papers published in indexed scientific journals (n=39), followed by Brazil (n=23), and France (n=22) (Figure 1). Universities obtained the highest number of granted projects (47%; n=34), followed by governmental health institutions (34%; n=25), research institutions (16%; n=12), and nongovernmental organizations (3%; n=2).

**Figure 2:**
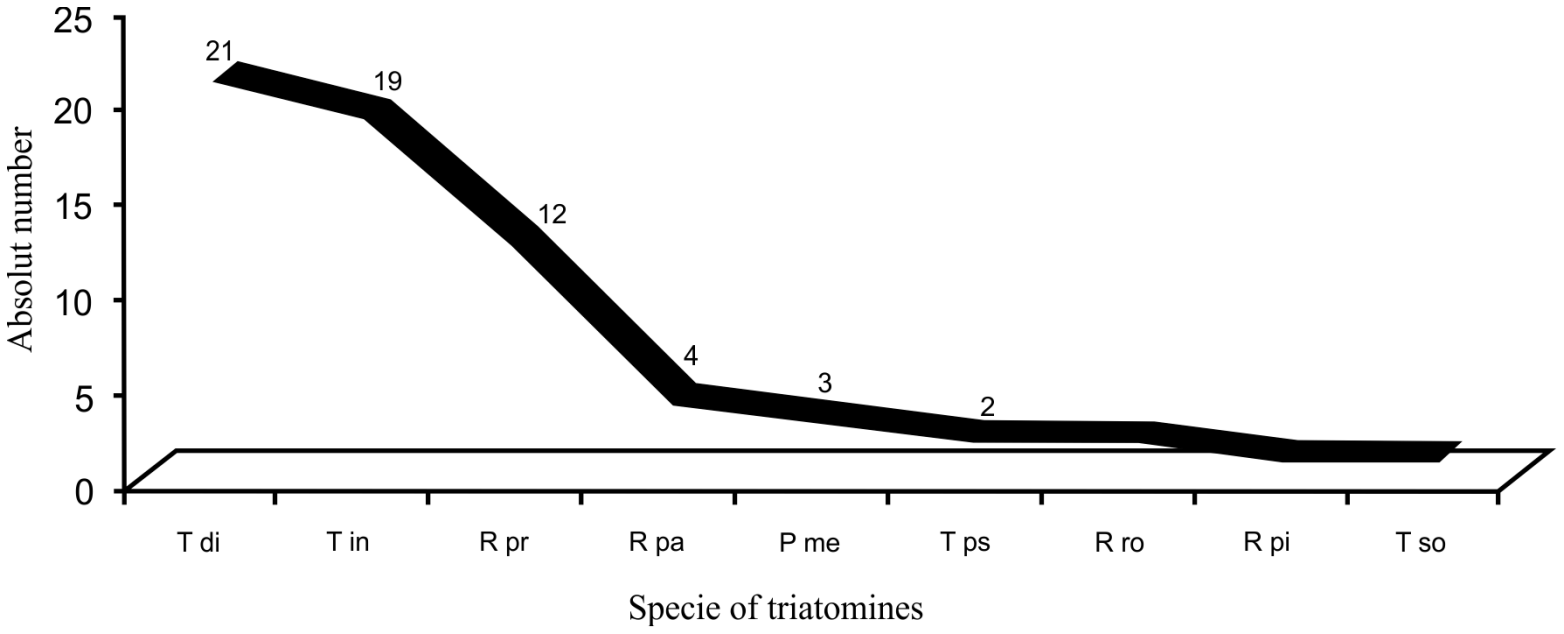
Species of triatomines studied by projects funded by CIRP/PAHO/TDR, 1997–2007 period. Legends: Tdi=*Triatoma dimidiata*; Tin=*T. infestans*; Rpr=*Rhodnius prolixus*; Rpa=*R. pallescens*; Pme=*Panstrongylus megistus*; Tps=*T. pseudomaculata*; Rro=*R. robustus*; Rpi=*R. pictipes*; Tso=*T. sordida*. Other species with one project funded: *T. vitticeps*, *T. maculata, T. longipennis*, *P. geniculatus*, *R. pictipes, R. ecuadoriensis*, and *R. colombiensis*.

The main topics addressed by the funded projects included the origin of reinfestation; vector resistance to insecticides and the establishment of a Latin American network for studies on vector control; chemical control (*e.g.*, substances as baits for trapping); cost-reduction/cost-effectiveness of insecticide interventions for control; risk maps and entomological surveillance; habitat suitability predictions; influence of sylvatic and peridomestic triatomines in domestic transmission; genetic and phenetic markers; population structure; primers design; validation of new criteria of cure; seroprevalence studies in children, blood banks, and general population; transmission mechanisms of *T. cruzi*; nonconventional flow-cytometric approaches; and a virtual atlas of Mexican triatomines. A total of 59 projects involved at least one vector in their studies, and the main triatomine species studied are shown in Figure 2.

### 2. Publication Databases Search

The CIRP/PAHO/TDR projects yielded 126 scientific articles and five book chapters, totaling 131 contributions published from 1997 to 2010. The average number of papers per year was one from 1997 to 1999 (n=3) and 11 from 2000 to 2010 (n=123). The highest and the lowest numbers of articles produced by a single project were 17 and one, respectively. The average time from the beginning of a project until the publication of the first related article was 3.4 years (range: one to eight years). The number of authors per article varied from one to 14, with an average of five coauthors per paper.

Of the 126 scientific articles, 120 were published by 31 PIs in 31 indexed journals of a total of 37, with an average five-year impact factor of 2.24 (Table 1). According to the of the Web of Knowledge databases, 84 of these 120 articles were cited in other articles, with an average of 38.3 citations (range: three to 125) per paper. The majority of the papers were written in English (n=112; 88.9%), followed by Spanish (n=8; 6.3%), and Portuguese (n=6; 4.8%). No scientific production was related to 31 CIRP/PAHO/TDR-funded projects (42.5%) involving 27 PIs. A summary of the results is shown in Table 2.

**Table 1:** Number of scientific articles derived from proposals to CIRP/PAHO/TDR, 1997–2007.

Journals^a^ indexed in the Web of Knowledge databases	ISSN	5-YIF	N
*Memorias do Instituto Oswaldo Cruz*	0074-0276	2.081	20
*Acta Tropica*	0001-706X	2.500	17
*Journal of Medical Entomology*	0022-2585	2.257	14
*American Journal of Tropical Medicine and Hygiene*	0002-9637	2.884	12
*Infection, Genetics & Evolution*	1567-1348	3.055	5
*Journal of Insect Physiology*	0022-1910	2.378	4
*Cadernos de Saúde Pública*	0102-311X	0.987*	4
*Physiological Entomology*	0307-6962	1.640	4
*PLOS Neglected Tropical Diseases*	e1935-2735	4.849	4
*Revista da Sociedade Brasileira de Medicina Tropical*	0037-8682	0.847	4
*Journal of Comparative Physiology A*	0340-7594	2.138	3
*Tropical Medicine & International Health*	1360-2276	2.967	3
*Anais da Academia Brasileira de Ciências*	0001-3765	1.201	2
*Biomedica*	0120-4157	0.442*	2
*Journal of Chemical Ecology*	0098-0331	2.551	2
*The Journal of Parasitology*	e1937-2345	1.365	2
*Journal of Immunological Methods*	0022-1759	2.322	2
*Medical & Veterinary Entomology*	0269-283X	2.210	2
*Applied Mathematical Modelling*	0307-904X	1.502	1
*Biological Rhythm Research*	0929-1016	0.668	1
*Bone Marrow Transplantion*	0268-3369	3.339	1
*Comparative Bioquemistry and Physiology A*	1095-6433	2.302	1
*Journal of Clinical Virology*	1386-6532	3.607	1
*Journal of Experimental Biology*	0022-0949	3.424	1
*Liver Transplantation*	1527-6465	3.595	1
*Parasites & Vectors*	1756-3305	2.140	1
*Parasitology*	0031-1820	2.530	1
*Pest Management Science*	1526-498X	2.358	1
*Reports in Public Health*	0033-3549	1.520	1
*Revista do Instituto de Medicina Tropical de São Paulo*	0036-4665	0.934*	1
*Vector-Borne and Zoonotic Disease*	1530-3667	2.757	1
*Entomotropica* a	1317-5262	-	2
*Boletin Chileno de Parasitología* a	0365-9402	-	1
*Círculo Médico de Salta* a	1514-6219	-	1
*Revista Entomología Mexicana* a	968839518-8	-	1
*Pan American Journal of Public Health* a	1020-4989	-	1
*Revista de Toxicología en Línea* a	1668-091X	-	1
Total			126

5-YIF=5-year impact factor, N=number of scientific articles published, ISSN=International Serial Standard Number,

a Journals not indexed in the Web of Knowledge databases.

* Without 5-year impact factor, values correspond to impact factor of 2010.

**Table 2:** Summary of main results for the projects funded by CIRP/PAHO/TDR about Chagas disease, 1997–2007.

Item	N
Projects approved	73
Countries involved	18
Principal Investigators (PI)	58
PIs who responded to the questionnaire*	29
Projects with additional information*	40
Articles published	126
Abstracts published⧫	125
Technical reports⧫	17
Extension activities⧫	27
Graduated students⧫	57
Specialization students⧫	6
Master students⧫	14
Ph.D. students⧫	26
Postdoc students⧫	4
Patents	2

* Data collection was completed with a questionnaire sent to the PI of each project in order to obtain detailed or extra information about the impact indicators of each project.

⧫Results provided by the in-depth survey.

The CIRP/PAHO/TDR projects yielded a mean of 1.8 scientific publications per project. Seventy-four (58.7%) of the scientific articles were coauthored by authors from different countries. The interaction among researchers was not high; 16 PIs of the 73 granted projects have some degree of synergetic collaboration.

**Figure 3:**
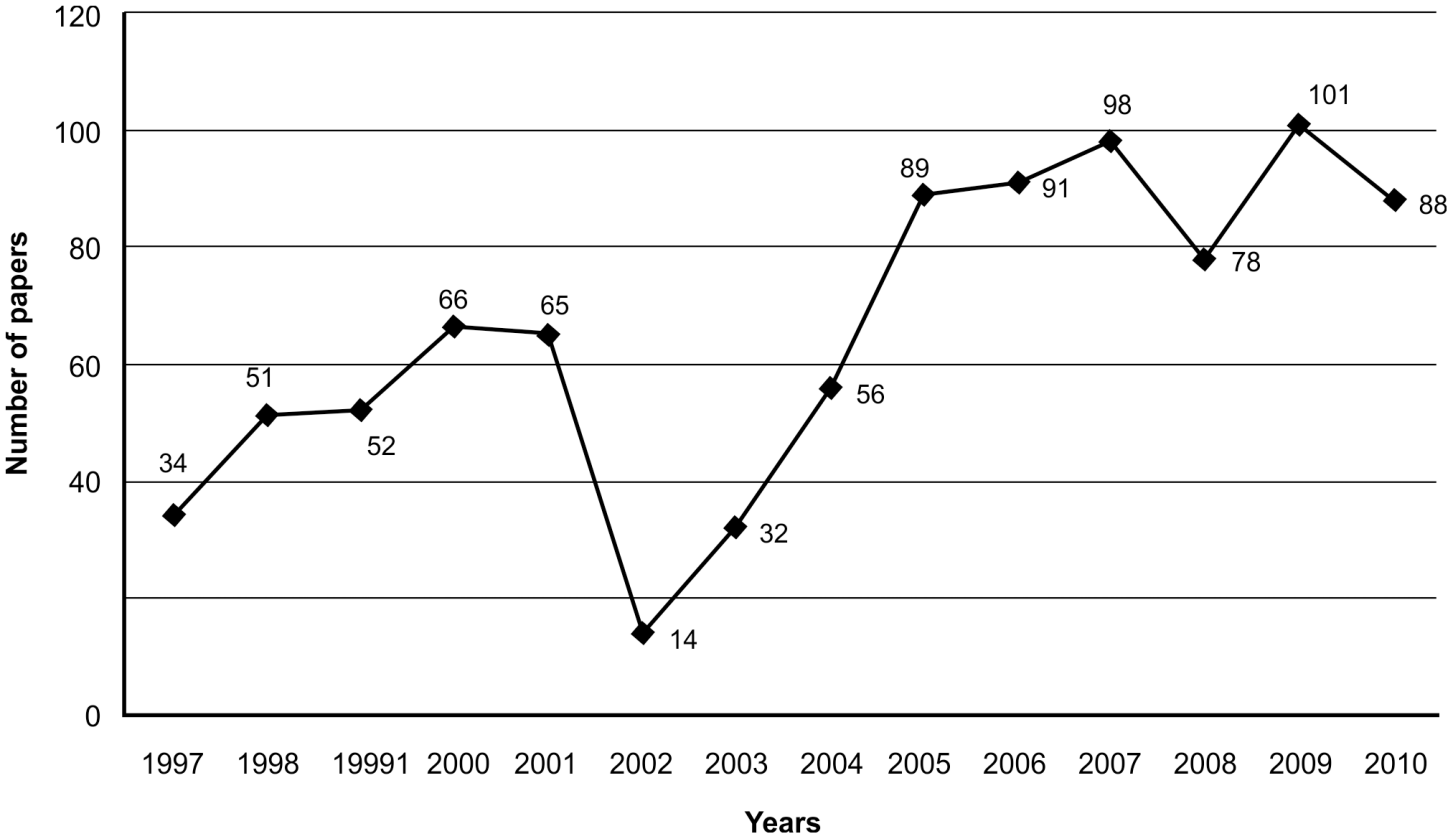
Number of studies identified and retrieved from PubMed using the parameter "triatom*," 1997–2010 period.

A total of 1,487 papers were retrieved searching PubMed using the term "triatom*." Of them, 915 (61.5%) were related to the research priorities of the CIRP/PAHO/TDR programme [13]. With this same search criterion, we retrieved a total of 117 papers derived from projects granted by CIRP/PAHO/TDR, accounting for 12.8% (117/915) of the total scientific publications for the study period. An increase in the number of published articles by year was observed, excepting the 2002–2004 period (Figure 3). The main language was English (n=779), followed by Portuguese (n=79), Spanish (n=55), and French (n=2); 68.5% (n=627) of the published articles were concentrated in 19 journals, with *Memorias do Instituto Oswaldo Cruz* having the most published articles (n=190) (Table 3).

**Table 3:** Journals with ten or more published papers about Chagas disease recorded in PubMed using the search term "triatom*."

Journal*	ISSN	N
*Memórias do Instituto Oswaldo Cruz*	*0074-0276*	190
*Revista da Sociedade Brasileira de Medicina Tropical*	0037-8682	58
*Journal of Medical Entomology*	0022-2585	57
*Acta Tropica*	0001-706X	53
*American Journal of Tropical Medicine and Hygiene*	0002-9637	51
*Cadernos de Saúde Pública*	0102-311X	29
*Infection, Genetics & Evolution*	1567-1348	25
*Parasitology Research*	0932-0113	20
*Medical & Veterinary Entomology*	0269-283X	19
*PLOS Neglected Tropical Diseases*	e1935-2735	16
*Anais da Academia Brasileira de Ciências*	0001-3765	15
*Biomedica*	0120-4157	15
*Experimental Parasitology*	0014-4894	14
*Revista do Instituto de Medicina Tropical de São Paulo*	0036-4665	12
*International Journal for Parasitology*	0020-7519	11
*Neotropical Entomology*	1519-566X	11
*Vector-Borne and Zoonotic Diseases*	1530-3667	11
*Parasitology*	0031-1820	10
*Transactions of the Royal Society of Tropical Medicine and Hygiene*	0035-9203	10
**Total**		**627**

N=number of scientific articles published, ISSN=International Serial Standard Number.

* Search period: 1997–2010.

Table S1 shows examples of studies funded by CIRP/PAHO/TDR summarizing their main result and their impact in Chagas control practice.

### 3. In-Depth Survey

**Figure 4:**
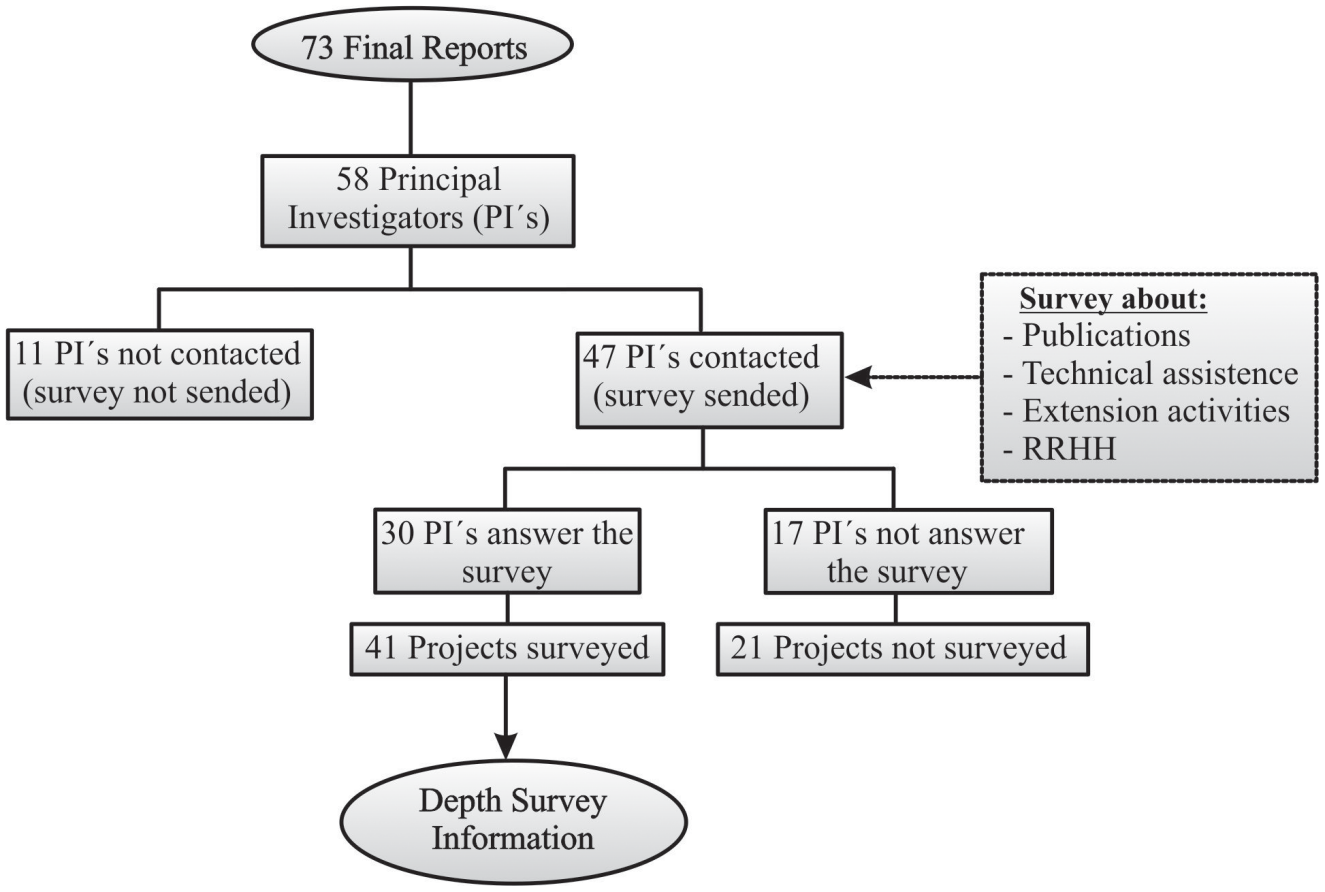
Flow diagram of the "in-depth survey" to Principal Investigators. CIRP/TDR/PAHO, 1997–2007 period.

Forty-seven of the 58 PIs could be contacted. Among them, 30 PIs (51.7%) of 41 projects (56%) (Figure 4) completed and returned the questionnaires.

The analysis of the questionnaires indicated that, on average, the scientific production per project was: 3.1 meeting abstracts (n=125), 2.7 graduated and post-graduate students (n=107), 0.4 technical reports (n=17), and 0.7 extension activities (n=27). In one single project, 750 health technicians were trained for carrying out entomo-epidemiological surveillance with the participation of the community. In addition, two patents were submitted related to chemical substances as baits for trapping triatomines: patent N° 012349/2003 in Brazil (National Institute of Industrial Propriety) by M. G. Lorenzo *et al.* and patent N° P000104304 in Argentina by A. Fontán *et al.*

CIRP/PAHO/TDR have organized and funded a series of training courses, including: a) Geographic Information Systems, spatial analysis and vector-borne diseases, with emphasis on Chagas disease (two courses with participants from ten countries of the Region); b) sampling and detection of sylvatic triatomines (two subregional courses, one in Bolivia and one in Guatemala with the participation of researchers and programme entomologists from South American countries and Central American countries and Mexico, respectively); c) evaluation of the cost-effectiveness of Chagas disease control interventions (two subregional courses, in Guatemala and Buenos Aires, respectively). In addition, the programme supported the organization of two meetings and the publication of their respective reports: a) decentralization and management of communicable diseases control in Latin America (http://www.paho.org/english/ad/dpc/cd/res-descentralizacion.pdf) and b) Scientific Working Group on Chagas Disease (http://www.who.int/tdr/publications/tdr-research-publications/reporte-enfermedad-chagas/en/index.html) (data not shown).

## Discussion

A huge body of scientific evidence has been gathered since the discovery of Chagas disease in 1909. Research has played an important role in the history of the control of Chagas disease, allowing a decrease of vectorial and transfusional transmission, and an interruption of *T. cruzi* transmission in some areas of the Region of the Americas [7], as well as the recognition of the limitations of interventions in some other areas [16]. Some of the research breakthroughs and control milestones were summarized in 1999 [7] and in a series of papers published during the celebration of the 100^th^ anniversary of the discovery of the disease [1], [2], [17].

International organizations such as PAHO/WHO, JICA, CIDA, MSF, WV, CI, DNDi, EC-FP7 Programme, and ECLAT, among others, have played an important role in accompanying the Chagas control initiatives in the Region of the Americas, as well as in supporting key research for the control and interruption of transmission in many countries of the Region. CIRP/PAHO/TDR has been one of many initiatives supporting research and capacity building in institutions of endemic countries that have permitted the acquisition of knowledge that contributed to the generation of evidence for vector control of Chagas disease [18]–[21], treatment of chronic Chagas disease [22], [23], and standardization and validation of diagnostic techniques [24]–[26], among others key research priorities.

During this period, the programme has supported 73 research projects on issues identified as research needs for Chagas disease control; they were carried out in endemic countries by researchers that in their majority were working in institutions located in these countries. These projects yielded 126 scientific articles published in peer-reviewed journals with a mean of 1.8 scientific publications per project. Most of the scientific publications originated from the Southern Cone countries, with Argentina and Brazil publishing, as expected, the highest number since they are countries with a high research production [27], [28].

The main language of publication was English, followed by Portuguese and Spanish, the official languages of most endemic countries. Similar results were obtained by others authors using search terms such as "Chagas disease" or "*Trypanosoma cruzi*" [27]. Our findings indicate that 70% (88/126) of the articles related to projects granted by CIRP/PAHO/TDR were published in journals that have as main scope tropical medicine, public health, and parasitology/entomology (data not shown), the Brazilian journal "*Memorias do Instituto Oswaldo Cruz*" being the one with the highest number of articles published. In a previous analysis of publications about Chagas disease [27], similar results were obtained.

In a previous bibliometric study of more than 850 articles and abstracts retrieved using the search term "triatomine," it was shown that only 13% of them dealt with control/surveillance interventions (search term: "Triatomin* AND control") [29], indicating that in general, and contrary to the expectations of most funding agencies, research efforts have little bearing on disease control. Using the same search criteria for the 1997–2008 period we found that 7.5% of the articles retrieved with the search term "Triatomin*") and 9.0% of those retrieved with "Triatomin* AND control" were derived from projects granted by CIRP/PAHO/TDR, representing a very high proportion of the papers retrieved.

In addition, our data show that 64% of the 73 projects funded by CIRP/PAHO/TDR responded to the research needs and were focused, as Table S1 summarizes, on issues such as the epidemiological study of the impact of the control measures, the updating of information on the incidence of infection in young age groups, the entomological study of nondomiciliated triatomines, the genetics of vector populations, the generation of information on possible emergence of insecticide resistance and the establishment of a network for monitoring the resistance of vectors to insecticides, the cost-effectiveness of control strategies, or the validation and standardization of PCR as a diagnostic test and a marker of treatment results.

The available information indicates that CIRP/PAHO/TDR has had not only an impact in the production of knowledge valuable for the control programmes, but also a pivotal role in creating a critical mass of researchers in Chagas disease at country level, with an average per project of 2.7 graduated and post-graduate students (n=107), 0.4 technical reports (n=17), 0.7 extension activities (n=27), and a high number of health technicians for carrying out the entomo-epidemiological surveillance with the participation of the community. Furthermore, the programme has supported training courses for researchers and capacity-building activities for the personnel of national control programmes and has promoted the collaboration between researchers and managers. Two workshops to standardize and validate cPCR [26] and qPCR as methods for Chagas disease diagnosis and surrogate markers of treatment results were also carried out (data not published). Networks of PIs working on surveillance of insecticides resistance as well as PCR have been created. In addition, the programme has supported research that generated the submission of patents related to chemical substances to be used as baits for trapping triatomines.

Most grantees reported being involved in other activities, including development of research proposals, teaching, supervision of doctoral and postdoctoral students, and serving in expert committees or as peer reviewers.

In 2005–2006, the structure and procedures of TDR were reviewed and restructured and a 2008–2013 plan including eleven business lines was established. The technical and scientific support for Chagas disease of CIRP/PAHO/TDR was included in this new plan, supporting the development and evaluation of improved and innovative vector control intervention such as community-based ecosystem management for the prevention of Chagas disease among other vector-borne diseases, the continuation of the initiative to standardize and validate molecular markers, the evaluation of treatment, as well as individual and or institutional capacity building at the country level. These initiatives are still in place and have already yielded and are still yielding evidence that will have an impact on practice for Chagas disease [30]–[37].

In conclusion, the results of this evaluation yield evidence supporting that CIRP/PAHO/TDR research on Chagas disease has supported research that generated valuable evidence for prevention and control of Chagas disease. Chagas control initiatives have achieved important reductions in the incidence of Chagas disease in many Latin American countries. However, there are still areas producing new cases by oral transmission [38], [39], by wild triatomines [40], in areas where the control activities have been not successful [41], and in areas with nondomiciliated transmission [42], with many challenges ahead for the prevention and control of Chagas disease. These challenges include the need for new and/or improved drugs, diagnostics tools, vector research, eco-epidemiological studies, and integrated and sustainable control strategies, among others. Some of these needs are part of the actual TDR and PAHO research agenda.

We agree with Hotez *et al.* [43] that Chagas disease demands imperative attention from health policy makers to prioritize and develop a comprehensive strategy for surveillance, prevention, and control, for which more research and financial support are needed, both being crucial to address the unfinished agenda.

**Box 1. Key Learning Points**

- Chagas disease is a potentially life-threatening disease transmitted by triatomine bugs (Hemiptera: Reduviidae) that is caused by *T. cruzi*, a parasite that infects an estimated 10 million people worldwide.
- Initiatives led by PAHO aiming at the control of vectorial and transfusional transmission have led to the interruption of the transmission of the parasite by *T. infestans* in many areas of the Southern Cone and Central America.
- CIRP/PAHO/TDR has contributed to the control of Chagas disease by supporting research of knowledge gaps and priority needs and training activities for the scientific and nonscientific community.
- The need of better diagnostic tools, including biomarkers for assessing treatment efficacy, and safe treatments, as well as the development of an integrated vector control strategy make a compelling case to complete the unfinished research agenda.
- Funding and political will is needed to sustain this work and achieve success in the challenges ahead.

**Box 2. Top 5 Papers**

1. Coura JR, Pinto Dias JC (2009) Epidemiology, control and surveillance of Chagas disease -100 years after its discovery. Mem Inst Oswaldo Cruz 104: 31–40.
2. Morel CM (1999) Chagas disease, from discovery to control - and beyond: history, myths and lessons to take home. Mem Inst Oswaldo Cruz 94: 3–16.
3. World Health Organization (2008) Chagas disease: control and elimination. Report of the Secretariat.
4. Gürtler RE, Diotaiuti L, Kitron U (2008) Commentary: Chagas disease: 100 years since discovery and lessons for the future. Int J Epidemiol 37: 698–701.
5. Abad-Franch F, Santos WS, Schofield JC (2010) Research needs for Chagas disease prevention. Acta Trop 115: 44–54.

## Supporting Information

Table S1 **Examples of Chagas disease research funded by CIRP/PAHO/TDR and their impact for control.**
(DOC)
