# Supplementary material for: A Scientometric Evaluation of the Chagas Disease Implementation Research Programme of the PAHO and TDR
Source: PLoS Negl Trop Dis. 2013 Nov 7;7(11):e2445. doi: 10.1371/journal.pntd.0002445 (PMC3820726; doi:10.1371/journal.pntd.0002445)
Supplement: Table S1 — Examples of Chagas disease research funded by CIRP/PAHO/TDR and their impact for control. (DOC) [file pntd.0002445.s001.doc]

| Principal Investigator and / or References/ or Grant Number | Initiatives* | Study Description | Main finding | Implication for control |
| --- | --- | --- | --- | --- |
| Noireau F | INCOSUR | Ecological study of *T. pseudomaculata* (Brazil). | *T. pseudomaculata* is found in trees and bird nests, but without preference for any particular tree species, but *T. juazeirensis* is rupicolous. Both species invade peridomestic structures but do not display a significant ability to colonize human dwellings and highly adaptable to different habitats, in the peridomestic area. | Provides new information about two candidates’ vectors from Brazil. |
| Picollo MI | INCOSUR | Evaluation of Insecticide sensitivity (Argentina). | High levels of resistance to the pyrethroid insecticides are detected in triatomines collected from northern Argentina. | Provided evidence to support the surveillance of *T. infestans* sensitivity to pyrethroid insecticides and inform adequate management strategies. |
| Rojas de Arias A | INCOSUR | Experimental field assay to evaluate the use of semiochemicals accessibility for early detection of triatomine when used in simple sticky baited traps (Paraguay). | Trap sensitivity observed was 0.64 ± 0.15 while in controls reached 0.13 ± 0.04. Therefore, detection sensitivity of the system infestation oscillated from 12.5% to 63.6%. Previously they report four components as attractants for triatomines (patent number see Results section). | Chemically-baited along with community involvement, could benefit triatomine surveillance in endemic areas. |
| Miles M | AMCHA | Vector ecology study (Amazon Region: Brazil/ Ecuador). | Local-scale (ecotope-level) variation emerges as the main driver of palm tree infestation risk in Amazonia, although soil fertility may also have an influence over large (sub-regional) geographic scales; infestation is similar in different landscape classes, including pristine forests, rural areas, and urban forest fragments. | Provide valuable information useful to improve vector surveillance and control strategies. |
| Arcos Teheran L | IPA | Seroprevalence in general population and blood banks (Ecuador). | Seroprevalence ranged from 0.9% to 11.3% and seropositives were detected among children <I0 years old indicating possible active transmission in at least 11 out of 29 communities studied. | Provides evidence of infection and transmission in an area where there was not enough information. |
| Davies C | IPA | Risk factors study for domestic *R. prolixus* infestation (Venezuela). | *R. prolixus* are significantly associated with proximity to high densities of *Attalea butyracea* palm trees. | Provide information valuable to develop adequate surveillance and control strategies. |
| Monroy C | IPCA | Eco-Epidemiology Study (Guatemala). | *T. dimidiata*, *R. prolixus* and *T. nitida* were collected and find infected by *T. cruzi* with 22 communities with natural infections rates of 13.8-20.6%. Sex ratio is proposed as an index to show the mobility of *T. dimidiata* in different populations. *T. dimidiata* is the main vector in ten departments, and *R. prolixus* in two departments. | Provides valuable evidence of rate of infection and sex ratio in the three species of triatomines. |
| Caceres L | IPCA | Evaluation of insecticide sensitivity of *R. pallescens* and *R. prolixus* (Panama). | I and V instar nymphs of *R. prolixus* and *R. pallescens* are susceptible to deltamethrin and lambdacyhalothrin insecticides. | Provide baseline information on *R. prolixus* and *R. pallescens* susceptibility to insecticides. |
| Dumontiel E | IPCA | Entomological assessment of Triatomine populations in Southern Belize. | Significant risk for autochthonous Chagas disease transmission in central and southern Belize. Suggest a pattern of seasonal infestation by non-domiciliated triatomines, closely related to *T. dimidiata* from Yucatan, Mexico. | Provides evidence of transmission in an area with little previous information. |
| Goubiere S | M | They set up a modeling framework to evaluate the cost-efficiency of mixed strategies (Mexico). | Different potential control strategies applied in specific spatial patterns shows that the combination of insect screens in outer zones and cleaning of peridomicile in the centre area reduce infestation by both sylvatic and peri-domiciliated vectors. | Provide evidence of cost effective control strategies. |
| Schijman A | AR | International interlaboratory study to select best PCR based procedures for detection of *T. cruzi* in human blood samples and experimental workshop for transference of selected procedures. | Transference of four PCR methods with acceptable performance for detection of *T. cruzi* in blood samples from chronic Chagas patients. See Schijman *et al* 2011 . | Led to harmonization of PCR related procedures reaching to a consensus regarding the best methods for DNA extraction, amplification and detection, interpretation of results and implementation of  quality controls  Improved molecular tool for  detection of *T. cruzi* |
| Schijman A [12] | AR | International workshop to transfer two Real time PCR methods using TaqMan technology in a multiplex format for accurate quantification of parasitic loads in human blood samples. | Analytical performance of both methods was established (reportable range, inclusivity and selectivity, precision and limits of detection and quantification). Application of the same methods in a same venue demonstrated the variability of parasitic loads in populations from different endemic regions and clinical and epidemiological scenarios. (See Duffy *et al* 2013 [12] and Ramirez JC *et al* 2013, in preparation). | Participants at the workshop have acquired the know-how to validate the above mentioned Real Time assays in their own clinical settings. |
| Grant 30755 | INCOSUR-IPA | Ongoing multicenter, randomized, double-blind, placebo-controlled clinical trial in patients (Argentina, Brazil, Colombia). | The study addresses an important clinical issue aimed to evaluate the effectiveness of Benznidazole in reducing overall parasite load in patients with Chronic Chagas disease, and if real-time qPCR is an appropriate method to quantify the parasite burden in these patients. | The final results of BENEFIT study will shed light on the role of trypanocidal therapy in preventing cardiac disease progression and death and on the sensibility of qPCR as marker of treatment efficacy. |

* Data obtained from Final Reports and/or publications. INCOSUR: *Iniciativa del Cono Sur para controlar y eliminar la enfermedad de Chagas*, AMCHA: *Iniciativa de los Países Amazónicos para la Vigilancia y Control de la Enfermedad de Chagas*, IPCA: *Iniciativa de los Países de América Central para el Control de la Transmisión Vectorial, Transfusional y la Atención Médica de la Enfermedad de Chagas*, IPA: *Iniciativa de los Países Andinos de Control de la Transmisión Vectorial y Transfusional de la Enfermedad de Chagas*, M= México, IC= International Collaboration, AR: American Region.

**References of Table S1:**

1. de la Fuente AL, Dias-Lima A, Lopes CM, Emperaire L, Walter A, et al. (2008) Behavioral plasticity of Triatominae related to habitat selection in northeast Brazil. J Med Entomol 45: 14-19.

2. Picollo MI, Vassena C, Santo Orihuela P, Barrios S, Zaidemberg M, et al. (2005) High resistance to pyrethroid insecticides associated with ineffective field treatments in Triatoma infestans (Hemiptera: Reduviidae) from Northern Argentina. J Med Entomol 42: 637-642.

3. Rojas de Arias A, Abad-Franch F, Acosta N, Lopez E, Gonzalez N, et al. (2012) Post-control surveillance of Triatoma infestans and Triatoma sordida with chemically-baited sticky traps. PLoS Negl Trop Dis 6: e1822.

4. Abad-Franch F, Ferraz G, Campos C, Palomeque FS, Grijalva MJ, et al. (2010) Modeling disease vector occurrence when detection is imperfect: infestation of Amazonian palm trees by triatomine bugs at three spatial scales. PLoS Negl Trop Dis 4: e620.

5. Black CL, Ocana-Mayorga S, Riner DK, Costales JA, Lascano MS, et al. (2009) Seroprevalence of Trypanosoma cruzi in rural Ecuador and clustering of seropositivity within households. Am J Trop Med Hyg 81: 1035-1040.

6. Sanchez-Martin MJ, Feliciangeli MD, Campbell-Lendrum D, Davies CR (2006) Could the Chagas disease elimination programme in Venezuela be compromised by reinvasion of houses by sylvatic Rhodnius prolixus bug populations? Trop Med Int Health 11: 1585-1593.

7. Monroy C, Rodas A, Mejia M, Rosales R, Tabaru Y (2003) Epidemiology of Chagas disease in Guatemala: infection rate of Triatoma dimidiata, Triatoma nitida and Rhodnius prolixus (Hemiptera, Reduviidae) with Trypanosoma cruzi and Trypanosoma rangeli (Kinetoplastida, Trypanosomatidae). Mem Inst Oswaldo Cruz 98: 305-310.

8. Caceres L, Rovira JR, Calzada J, Saldana A (2011) [Evaluation of the toxic activity of the pyrethroid insecticides deltamethrin and lambdacyhalothrin in two Panamanian field populations of Rhodnius pallescens (Hemiptera: Reduviidae)]. Biomedica 31: 8-14.

9. Polonio R, Ramirez-Sierra MJ, Dumonteil E (2009) Dynamics and distribution of house infestation by Triatoma dimidiata in central and southern Belize. Vector Borne Zoonotic Dis 9: 19-24.

10. Barbu C, Dumonteil E, Gourbiere S (2009) Optimization of control strategies for non-domiciliated Triatoma dimidiata, Chagas disease vector in the Yucatan Peninsula, Mexico. PLoS Negl Trop Dis 3: e416.

11. Schijman AG, Bisio M, Orellana L, Sued M, Duffy T, et al. (2011) International study to evaluate PCR methods for detection of Trypanosoma cruzi DNA in blood samples from Chagas disease patients. PLoS Negl Trop Dis 5: e931.

12. Duffy T, Cura CI, Ramirez JC, Abate T, Cayo NM, et al. (2013) Analytical Performance of a Multiplex Real-Time PCR Assay Using TaqMan Probes for Quantification of Trypanosoma cruzi Satellite DNA in Blood Samples. PLoS Negl Trop Dis 7: 17.

13. Marin-Neto JA, Rassi A, Jr., Morillo CA, Avezum A, Connolly SJ, et al. (2008) Rationale and design of a randomized placebo-controlled trial assessing the effects of etiologic treatment in Chagas' cardiomyopathy: the BENznidazole Evaluation For Interrupting Trypanosomiasis (BENEFIT). Am Heart J 156: 37-43.

14. Marin-Neto JA, Rassi A, Jr., Avezum A, Jr., Mattos AC, Rassi A, et al. (2009) The BENEFIT trial: testing the hypothesis that trypanocidal therapy is beneficial for patients with chronic Chagas heart disease. Mem Inst Oswaldo Cruz 104 Suppl 1: 319-324.
